# Effect of subacute poisoning with lambdacyhalothrin on vascular endothelial growth factor 2 receptor in mice kidneys

**DOI:** 10.1186/s12882-022-02828-1

**Published:** 2022-05-28

**Authors:** Barbara Nieradko-Iwanicka, Maciej Rutkowski

**Affiliations:** 1 Department of Hygiene and Epidemiology, Medical University of Lublin, Chodzki 7 Street, 20-093 Lublin, Poland; 2 Military Medical Institute, Szaserów 128 Street, 04-141 Warszawa, Poland

**Keywords:** Lambdacyhalothrin, Vascular endothelial growth factor 2 receptor, Nuclear factor κB, Nephrotoxicity

## Abstract

**Background:**

Pyrethroids are used for insect control. They act on voltage-gated sodium channels in neurons. Lambdacyhalothrin (LCH) is a type II pyrethroid producing choreoatetosis and salivation in rodents. Vascular endothelial growth factor (VEGF) expression in the kidney is high in the glomerular podocytes and kidney tubules. VEGF receptor 2 (VEFGR2) is the main mediator in angiogenesis and it regulates blood vessel permeability. Lack of VEGF in podocytes impairs filtration. The nuclear factor κB (NFκB) is widely known as an important mediator of inflammation.

The aim of the study was to check if subacute oral intoxication with 0.1LD_50_ of LCH affects kidney size, function and VEGFR2 and NFκB in mice kidneys.

**Methods:**

A total of 32 Albino Swiss mice was used: females controls, males controls, females receiving 2 mg/kg LCH, males receiving 2 mg/kg LCH orally for 7 days. On day 8 animals were sacrificed, blood and kidneys were obtained. Kidney mass was determined, creatinine concentration was measured in blood sera, VEGFR2 and NFκB in kidney homogenate supernatant with ELISA kit.

**Results:**

There was no statistically significant differences in kidney mass, creatinine concentration in blood sera nor NFκB but mean VEGFR2 concentration in the kidneys of females exposed to LCH was 128.01 ng/ml and showed statistically significant difference in comparison with control females. There was no statistically significant difference between VEGFR2 concentration in the kidneys of males exposed to LCH and control males.

**Conclusion:**

The VEGFR2 increases in the course of LCH intoxication in females probably due to the protective effect of oestrogens.

## Background

Pyrethroids are commonly used for insect pest control. Chemically, they are esters of pyrethrinic or chrysanthemic acid and alcohols. They are widely used for crop protection and recommended as biocides and repellents against human and animal ectoparasites, to treat scabies and lice, to prevent malaria, to control ticks carrying pathogenic bacteria and viruses, indoors to fight ants, houseflies, mosquitoes and cockroaches [1–3]. Pyrethroids act mainly on the voltage-gated sodium channels in neurons leading to symptoms of neurotoxicity in target organisms [4]. Lambdacyhalothrin (LCH) is a type II pyrethroid containing the α-cyano moiety [5]. LCH is approved for use in the European Union on the basis of the permit of April 1, 2016, issued after another evaluation of the substance in accordance with the provisions of Directive 2009/128 / EC. This permit also specifies to which plants intended for food purposes it may be used. In Poland LCH is widely used for protection of potato, oilseed rape, wheat, barley, maize, apple trees, onions, cabbage, aronia, hop, common linen and hemp from pest insects. As it is widely used indoors and outdoors for pest control there are concerns about its toxicity for humans as non-target organisms [6] even though mammals are over 2000 times less sensitive to pyrethroids than insects [7]. The sodium channels in insects are more sensitive to pyrethroids than in mammals. Insects have lower body temperature and smaller body size than mice, rats or humans which metabolize the xenobiotics in their livers to low-toxic metabolites [7]. In mammals type II pyrethroids (including LCH) produce choreoatetosis and salivation interacting not only with the sodium channels, but also with calcium and chloride channels [5] and gamma-aminobutyric acid receptors [8], they can also produce seizures [7]. The oral LD_50_ of LCH for mice is 19.9 mg/kg [6]. Previous studies focused mainly on neurotoxicity of pyrethroids [7, 8], few on nephrotoxicity, immunotoxicity, hepatotoxicity and effect of fertility [9].

Vascular endothelial growth factor receptor 2 (VEGFR2) is the main mediator in angiogenesis and regulates blood vessel permeability. VEGFRs have their own tyrosine receptors [10, 11].VEGF expression in the kidney is high in glomerular podocytes as well as in kidney tubules [12–15]. VEGFRs are present in endothelial cells in glomeruli and in capillary blood vessels around the tubules [16, 17]. In physiological conditions constant expression of VEGF takes place in podocytes and endothelial cells which means that VEGF molecules migrate in opposite direction that glomerular filtration in order to get bound to their receptors. Lack of VEGF in podocytes leads to loss of fenestration of endothelium, which impairs filtration [18, 19] and may lead to development of kidney diseases. This confirms the crucial role of VEGFR in proper regulation of kidney filtration.

Originally the nuclear factor κB (NFκB) was described as a nuclear protein in the B lymphocytes. Its’ role is to regulate the expression of the immunoglobulin κ light chain gene [20, 21]. Subsequently, it was found that NFκB was a ubiquitous transcription factor mediating the signal-induced expression of a number of genes involved in many biological processes: immune response, inflammation, cell growth, survival and death. The NFκB is an important mediator of inflammation. However its role in the inflammatory processes in different organs is not fully understood [22].

The aim of the study was to check if oral subacute poisoning with 0.1LD_50_ of LCH affects kidney size, function and the level of VEGFR2 and NFκB in mice kidneys.

## Methods

The project of the experiment was accepted by The Local Ethical Committee in Lublin (Lokalna Komisja Etyczna do spraw Doświadczeń na Zwierzętach przy Uniwersytecie Medycznym w Lublinie), Poland (69/2015). All methods were carried out in accordance with relevant guidelines and regulations at the Centre for Experimental Medicine (CEM) at The Medical University of Lublin, Poland. The experiment was conducted in standard laboratory conditions (12 h light/dark cycle, temperature 21-22 °C, 55-60% relative air humidity). The animals had access to water (UV sterilized) and feed. The feed for rodents was purchased from Altromin International (Lage, Germany). A total of 32 young adult mice (Albino Swiss) was used. There were 16 non gravid females and 16 young adult males. The animals were 6 weeks of age. They were bred at CEM. The herd originated from Charles River Laboratories (Cologne, Germany). The females were randomly divided into 2 groups of 8: controls receiving canola oil by gavage and females receiving 2 mg/kg LCH by the same route. Males were also randomly divided into 2 groups of 8: the control group receiving canola oil by gavage and males receiving 2 mg/kg LCH. The LCH was purchased form Organic Chemistry Institute (Annopol 6, 03-236 Warsaw, Poland). It was dissolved in canola oil (ZT ‘Kruszwica’, Kruszwica, Poland), and administered daily by gavage for 7 consecutive days. On day 8 the animals were euthanized. They were decapitated with a guillotine without former anesthesia. We didn’t want the anesthetics to affect the biochemical parameters. The blood and kidneys were obtained. Kidney mass was recorded. Creatinine was measured with Erba Mannheim XL-60 biochemistry analyzer (Mannheim, Germany). The kidneys were homogenized with a mechanical blender MPW-120 (MPW Med. Instruments, Warsaw, Poland) in 0.1 mol buffer of Tris-HCl, of pH 7.4. For the analyses 0.5 g of kidney tissue was blended in 5 ml of the buffer. Next the homogenates were centrifuged for 15 min (5000×g) twice (Sigma1-6P centrifuge, Polygen, Engelwood, NY, USA was used). The supernatant was used for measuring VEGFR2 and NFκB with Enzyme Linked Immunoabsorbent Assay tests. The kits were purchased from manufacturer (Cloud-Clone Corp. Katy, TX, USA). The results were analyzed with IBM SPSS Statistics (v. 21) (Statsoft Sp. z o. o. Cracow, Poland). The Mann–Whitney test was used to assess the difference between two groups; *p* < 0.05 was considered statistically significant.

## Results

The concentration of creatinine was (mean ± SD) 0.2 ± 0.0 mg/dl in control females and males. In the groups exposed to LCH it was 0.2 ± 0.04 in females and 0.2 ± 0.03 in males and it did not differ significantly from controls. Kidney mass was (mean ± SD) 0.16 ± 0.02 g in control females and males, 0.18 ± 0.01 g in females intoxicated with LCH and 0.19 g ± 0.02 g in experimental males. There was no significant differences among the groups.

The VEGFR2 concentration in the kidneys of females exposed to LCH was (mean ± SD) 128.01 ± 36.13 ng/ml and was statistically significantly higher than in control females 84.28 ± 2649 ng/ml (*p* < 0,05). There was no statistically significant difference between VEGFR2 concentration in the kidneys of males exposed to LCH 170.16 ± 58.3 ng/ml if compared with control males 170.61 ± 37.03 ng/ml. The NFκB in females exposed to LCH was 3.63 ± 1.82 ng/ml and in males after LCH it was 4.04 ± 1.35 ng/ml. The values did not significantly differ from controls (Table 1).

**Table 1:** The influence of LCH on kidney mass, serum creatinine, VEGFR2 and NFκB concentration in mice kidneys

Group	Creatinine [mg/dl] (mean ± SD)	Kidney mass [g] (mean ± SD)	VEGFR2 [ng/ml] (mean ± SD)	NFκB [ng/ml] (mean ± SD)
Control females (*n* = 8)	0.2 ± 0.0	0.16 ± 0.02	84.28 ± 2649	3.27 ± 0.97
Females receiving 2 mg/kg LCH for 7 days (*n* = 8)	0.2 ± 0.04	0.18 ± 0.01	128.01 ± 36.13*	3.63 ± 1.82
Control males (*n* = 8)	0.2 ± 0.0	0.16 ± 0.02	170.61 ± 37.03	4.09 ± 0.06
Males receiving 2 mg/kg LCH for 7 days (*n* = 8)	0.2 ± 0.03	0.19 g ± 0.02	170.16 ± 58.3	4.04 ± 1.35

^*^p < 0.05 vs controls

## Discussion

Pyrethroids, including LCH, are metabolized in the liver by cleavage of the ester linkage, followed by conversion to 3-phenoxybenzoic acid (3-PBA) and cyclopropanecarboxylic acid, which are further metabolized by hydroxylation and glucuronidation and excreted mainly with urine [23]. The kidneys contribute to the maintenance of the body’s homeostasis thanks to the excretion of these unnecessary metabolic products. Concentrating urine in the tubular fluid also increases the concentration of xenobiotics in it. Renal transport and the accumulation and biotransformation of pyrethroid metabolites contribute to the susceptibility of the kidneys to damage by this group of xenobiotics. In a population study published by Wielgomas et al. for the first time in Poland, assessed the exposure to synthetic pyrethroids in preschool and school children and their parents living in urban and rural areas. The study examined urine samples collected from 374 residents of northern regions of Poland, assessing the concentration of the main pyrethroid metabolites, which are mainly excreted by the kidneys. All tested metabolites were detected in higher concentrations in inhabitants of rural areas. 3-PBA was detected in 77.4% of samples taken from urban residents and in as much as 93.8% of samples collected from rural residents. The situation was similar with the other metabolites [24]. The study clearly indicates high probability of intoxication with pyrethroids used in agriculture and household.

Our results show that kidney function was not significantly impaired in the course of subacute poisoning with 0.1 LD_50_ of LCH. The kidney size was not increased in males and females intoxicated with the pesticide which may result from low dose of pyrethroid used. We aimed to mimic the possible human exposure to low doses of LCH with plant foods or water. Unfortunately, due to lack of finances, we couldn’t perform histopathologic examination of the kidneys nor other internal organs nor hormone measurement. However the animals used in our study were young adults. Therefore we expected all of the female animals to have high oestrogen levels and males to have high testosterone.

Fedeli et al. in their experiment with permethrin showed, that it accelerated the aging process by decreasing the glomerular filtration rate and impairing sodium excretion [25]. The difference may result from the fact that permethrin has different structure than LCH, as permethrin is a type I pyrethroid.

Abdel- Daim et al. administered a type II pyrethroid deltamethrin at the dose of 2 mg/kg orally for 4 weeks to rats and proved its nephrotoxicity. Urea, uric acid and creatinine concentrations were increased [26]. Martinez et al. showed that LCH at the dose of 4 and 8 mg/kg induced high expression of proinflmmatory cytokines in rats [27].

There is data that VEGFR2 blockade improves kidney function in diabetic nephropathy [28]. Despite proinflammtory effect of the pyrethroid, kidneys of animals used in our study were able to regenerate thanks to high expression of VEGFR2. The effect was well visible in females.

The role of VEGF in maintaining microcirculation in the kidneys has been investigated in a number of animal models. In the ischemia-reperfusion model of renal injury, the production of VEGF in the kidney is not increased, but there is a redistribution of already produced VEGF to the kidneys and increased expression of VEGFR-2 mRNA [29, 30]. Ischemia and reperfusion lead to oxidative stress in the kidneys [31] and so does intoxication with pyrethroids [32]. There are reports suggesting that mesenchymal stem cells act to protect kidney damage in an ischemia-reperfusion model not through cell regeneration, but through paracrine mechanisms. VEGF is one of the most important factors in these mechanisms [33]. Studies in rats have demonstrated chronic renal dysfunction and a decrease in the number of capillaries in the renal glomeruli and the periurethral space associated with a decrease in renal VEGF expression [34]. The administration of VEGF in this model had a protective effect on the vascular endothelium and allowed the inhibition of the progression of renal dysfunction, as well as scarring of tissue damage regardless of blood pressure, proteinuria or macrophage infiltration [35]. It has been proved that VEGF transcription is regulated by estrogens, the secretion of which is mediated by stimulation of the estrogen receptor [36]. Estrogens showed a protective effect on the residual kidney of rats, suggesting that it might be via the regulation of VEGF expression [37]. In this situation, it is possible that females that produce more estrogen than males have a higher VEGFR.

The NFκB can be activated by many factors. It is worth noting that the processes in which it is involved may also affect the metabolism of xenobiotics and the activity of liver enzymes [38, 39]. NFκB binding sites have been identified in the promoters of the genes of some xenobiotic metabolizing enzymes [40]. Martínez et al. used LCH at a dose of 4 mg/kg body weight of rats and it increased the expression of NFκB specific mRNA by 1.37 times [27]. In our experiment, however, there was no significant increase of NFκB in kidney tissue after intoxication with LCH neither in females nor in males. It was probably due to the fact that the dose of LCH used in our experiment was much lower than in the study of Martínez et al.

Traditional markers of acute kidney damage (proteinuria, urinary albumin excretion, hematuria, bacteriuria, rolleruria, oliguria or anuria, alteration of the fractional excretion of sodium in the urine, increased values of renal excretory function parameters, disturbances in water-electrolyte and acid-base balance, imaging and hematology parameters) are most often clinically expressed only a few days after the action of the factor damaging the kidneys. In this experiment, according to the protocol the animals were decapitated on day 8 and it was impossible to monitor these parameters over long time. However there are new biomarkers of acute renal injury: cystatin C, neutrophil galatinase associated lipocalin (NGAL), interleukin-18, kidney injury molecule-1. The procalcitonin is helpful in the diagnosis of septic renal injury. Unfortunately neither the classic measurement of urinary albumin excretion nor the novel biomarkers of kidney damage were included in the study protocol as it focused on the NFκB and VEGFR2.

## Conclusion

The VEGFR2 increases in the course of LCH intoxication in females probably due to the protective effect of oestrogens. Nevertheless, preparations containing LCH should be used with caution only for registered uses and in accordance with the manufacturer’s instructions.

## Data Availability

All data generated or analysed during this study are included in this published article.

## Abbreviations

LCH: Lambdacyhalothrin
VEGF: Vascular endothelial growth factor
VEGF2: Vascular endothelial growth factor receptor 2
LD50: Lethal dose for 50% of the exposed population
NFκB: Nuclear factor κB
SD: Standard deviation
3-PBA: 3-phenoxybenzenoic acid

## References

[1] Matsuo N (2019). Discovery and development of pyrethroid insecticides. Proc Jpn Acad Ser B Phys Biol Sci.

[2] Feo ML, Eljarrat E, Manaca MN, Dobaño C, Barcelo D, Sunyer J, Alonso PL, Menendez C, Grimalt JO (2012). Pyrethroid use-malaria control and individual applications by households for other pests and home garden use. Environ Int.

[3] Tang W, Wang D, Wang J, Wu Z, Li L, Huang M, Xu S, Yan D (2018). Pyrethroid pesticide residues in the global environment: an overview. Chemosphere..

[4] Field LM, Davies TGE, O'Reilly AO, Williamson AO, Wallace BA (2017). Voltage-gated sodium channels as targets for pyrethroid insecticides. Eur Biophys J.

[5] Lu Q, Sun Y, Ares I, Anadón A, Martínez M, Martínez-Larrañaga MR, Yuan Z, Wang X, Martínez MA (2019). Deltamethrin toxicity: a review of oxidative stress and metabolism. Environ Res.

[6] National Pesticide Informatin Center. 2001 http://npic.orst.edu/factsheets/archive/l_cyhalotech.pdf Accessed 15 Sept 2020.

[7] Mohammadi H, Ghassemi-Barghi N, Malakshah O, Ashari S (2019). Pyrethroid exposure and neurotoxicity: a mechanistic approach. Arh Hig Rada Toksikol.

[8] Akelma H, Kilic ET, Salik F, Bicak EA, Yektas A (2019). Pyrethroid intoxication: a rare case report and literature review. Niger J Clin Pract.

[9] Chrustek A, Hołynska-Iwan I, Dziembowska I, Bogusiewicz A, Wróblewski M, Cwynar A, Olszewska-Słonina D (2018). Current research on the safety of pyrethroids used as insecticides. Medicina (Kaunas).

[10] Apte RS, Chen DS, Ferrara N (2019). VEGF in signaling and disease: beyond discovery and development. Cell..

[11] Logue OC, McGowan JW, George EM, Bidwell GL (2016). Therapeutic angiogenesis by vascular endothelial growth factor supplementation for treatment of renal disease. Curr Opin Nephrol Hypertens.

[12] Rana P, Pritchard KI, Kerbel R (2017). Plasma vascular endothelial growth factor as a predictive biomarker: door closed?. Eur J Cancer.

[13] Mandai H, Omori K, Yamamoto D, Tsumura T, Murota K, Yamamoto S, Mitsudo K, Ibaragi S, Sasaki A, Maeda H, Takashiba S, Suga S (2014). Synthetic (+)-terrein suppresses interleukin-6/soluble interleukin-6 receptor induced-secretion of vascular endothelial growth factor in human gingival fibroblasts. Bioorg Med Chem.

[14] Mandal K, Uppalapati M, Ault-Riché D, Kenney J, Lowitz J, Sidhu SS, Kent SB (2012). Chemical synthesis and X-ray structure of a heterochiral {D-protein antagonist plus vascular endothelial growth factor} protein complex by racemic crystallography. Proc Natl Acad Sci U S A.

[15] Stewart MW (2013). Aflibercept (VEGF trap-eye) for the treatment of exudative age-related macular degeneration. Expert Rev Clin Pharmacol.

[16] Kikuchi R, Stevens M, Harada K, Oltean S, Murohara T (2019). Anti-angiogenic isoform of vascular endothelial growth factor-a in cardiovascular and renal disease. Adv Clin Chem.

[17] Roskoski R (2017). Vascular endothelial growth factor (VEGF) and VEGF receptor inhibitors in the treatment of renal cell carcinomas. Pharmacol Res.

[18] Kikuchi R, Yasuda Y, Nakatochi M, Shibata Y, Hara T, Suzuki A, Imaizumi T, Suzuki S, Ishii H, Takeshita K, Matsushita T, Maruyama S, Murohara T (2017). Urinary and circulating levels of the anti-angiogenic isoform of vascular endothelial growth factor-a in patients with chronic kidney disease. Clin Chim Acta.

[19] Eremina V, Sood M, Haigh J, Nagy A, Lajoie G, Ferrara N, Gerber HP, Kikkawa Y, Miner JH (2003). Glomerular-specific alterations of VEGF-A expression lead to distinct congenital and acquired renal diseases. J Clin Invest.

[20] Sen R, Baltimore D (1986). Inducibility of kappa immunoglobulin enhancer-binding protein Nf-kappa B by a posttranslational mechanism. Cell.

[21] Sen R, Baltimore D (1986). Multiple nuclear factors interact with the immunoglobulin enhancer sequences. Cell.

[22] Schroppel, He JC (2006). Expression of toll-like receptors in the kidney: their potential role beyond infection. Kidney Int.

[23] Hedges L, Brown S, MacLeod AK, Vardy A, Doyle E, Song G, Moreau M, Yoon M, Osimitz TG, Lake BG (2019). Metabolism of deltamethrin and cis- and trans-permethrin by human expressed cytochrome P450 and carboxylesterase enzymes. Xenobiotica.

[24] Wielgomas B, Piskunowicz M (2013). Biomonitoring of pyrethroid exposure among rural and urban populations in northern Poland. Chemosphere.

[25] Fedeli D, Carloni M, Nasuti C, Gambini A, Scocco V, Gabbianelli R (2013). Early life permethrin exposure leads to hypervitaminosis D, nitric oxide and catecholamines impairment. Pestic Biochem Physiol.

[26] Abdel-Daim MM, El-Ghoneimy A (2015). Synergistic protective effects of ceftriaxone and ascorbic acid against subacute deltamethrin induced nephrotoxicity in rats. Ren Fail.

[27] Martínez MA, Ares I, Rodríguez JL, Martínez M, Roura-Martínez D, Castellano V, Lopez-Torres B, Martínez-Larrañaga MR, Anadón A (2018). Pyrethroid insecticide lambda-cyhalothrin induces hepatic cytochrome P450 enzymes, oxidative stress and apoptosis in rats. Sci Total Environ.

[28] Lavoz C, Rodrigues-Diez RR, Plaza A, Carpio D, Egido J, Ruiz-Ortega M, Mezzano S (2020). VEGFR2 blockade improves renal damage in an experimental model of type 2 diabetic nephropathy. J Clin Med.

[29] Kanellis J, Mudge SJ, Fraser S, Katerelos M, Power DA (2000). Redistribution of cytoplasmic VEGF to the basolateral aspect of renal tubular cells in ischemia-reperfusion injury. Kidney Int.

[30] Kanellis J, Paizis K, Cox AJ, Stacker SA, Gilbert RE, Cooper ME, Power DA (2002). Renal ischemia-reperfusion increases endothelial VEGFR-2 without increasing VEGF or VEGFR-1 expression. Kidney Int.

[31] Aboutaleb N, Jamali H, Abolhasani M, Pazoki TH (2019). Lavender oil (Lavandula angustifolia) attenuates renal ischemia/reperfusion injury in rats through suppression of inflammation, oxidative stress and apoptosis. Biomed Pharmacother.

[32] Nieradko-Iwanicka B, Borzęcki A (2015). Subacute poisoning of mice with deltamethrin produces memory impairment, reduced locomotor activity, liver damage and changes in blood morphology in the mechanism of oxidative stress. Pharmacol Rep.

[33] Tögel F, Weiss K, Yang Y, Hu Z, Zhang P, Westenfelder C (2007). Vasculotropic, paracrine actions of infused mesenchymal stem cells are important to the recovery from acute kidney injury. Am J Physiol Ren Physiol.

[34] Kang DH, Hughes J, Mazzali M, Schreiner GF, Johnson RJ (2001). Impaired angiogenesis in the remnant kidney model: II. Vascular endothelial growth factor administration reduces renal fibrosis and stabilizes renal function. J Am Soc Nephrol.

[35] Kang DH, Joly AH, Oh SW, Hugo C, Kerjaschki D, Gordon KL, Mazzali M, Jefferson JA, Hughes J, Madsen KM, Schreiner GF, Johnson RJ (2001). Impaired angiogenesis in the remnant kidney model: I. potential role of vascular endothelial growth factor and thrombospondin-1. J Am Soc Nephrol.

[36] Hyder SM, Nawaz Z, Chiappetta C, Stancel GM (2000). Identification of functional estrogen response elements in the gene coding for the potent angiogenic factor vascular endothelial growth factor. Cancer Res.

[37] Kang DH, Yu ES, Yoon KI, Johnson R (2004). The impact of gender on progression of renal disease: potential role of estrogen-mediated vascular endothelial growth factor regulation and vascular protection. Am J Pathol.

[38] Aitken AE, Richardson TA, Morgan ET (2006). Regulation of drug-metabolizing enzymes and transporters in inflammation. Annu Rev Pharmacol Toxicol.

[39] Morgan ET (2001). Regulation of cytochrome p450 by inflammatory mediators: why and how?. Drug Metab Dispos Biol Fate Chem.

[40] Morgan ET, Li-Masters T, Cheng PY (2002). Mechanisms of cytochrome P450 regulation by inflammatory mediators. Toxicology.

